# Biomarkers for Response of Melanoma Patients to Immune Checkpoint Inhibitors: A Systematic Review

**DOI:** 10.3389/fonc.2017.00233

**Published:** 2017-09-27

**Authors:** Charissa A. C. Jessurun, Julien A. M. Vos, Jacqueline Limpens, Rosalie M. Luiten

**Affiliations:** ^1^Department of Dermatology and Netherlands Institute for Pigment Disorders, Academic Medical Center, University of Amsterdam, Amsterdam, Netherlands; ^2^Medical Library, Academic Medical Center, University of Amsterdam, Amsterdam, Netherlands

**Keywords:** immune checkpoint inhibitors, predictive biomarkers, melanoma, immune response, PD-1, PD-L1, CTLA-4

## Abstract

**Background:**

Immune checkpoint inhibitors (ICIs), targeting CTLA-4 or PD-1 molecules, have shown impressive therapeutic results. However, only 20–40% of advanced melanoma patients have durable responses to ICI, and these positive effects must be balanced against severe off-target immune toxicity and high costs. This urges the development of predictive biomarkers for ICI response to select patients with likely clinical benefit from treatment. Although many candidate biomarkers exist, a systematic overview of biomarkers and their usefulness is lacking.

**Objectives:**

Here, we systematically review the current literature of clinical data of ICI treatment to provide an overview of candidate predictive biomarkers for ICI in melanoma patients.

**Methods:**

To identify studies on biomarkers for clinical response or survival to ICI therapy in melanoma patients, we performed a systematic search in OVID MEDLINE and retrieved 429 publications, of which 67 met the eligibility criteria.

**Results:**

Blood and genomic biomarkers were mainly studied for CTLA-4 ICI, while tumor tissue markers were analyzed for both CTLA-4 and PD-1 ICI. Blood cytology and soluble factors correlated more frequently to overall survival (OS) than to response, indicating their prognostic rather than predictive nature. Systemic T-cell response and regulation markers correlated to response, but progression-free survival or OS were not analyzed. Tumor tissue analyses revealed response correlations with mutational load, neoantigen load, immune-related gene expression, and CD8+ T-cell infiltration at the invasive margin. The predictive value of PD-L1 varied, possibly due to the influence of T-cell infiltration on tumor PD-L1 expression. Genomic biomarker studies addressed CTLA-4 and other immune-related genes.

**Conclusion:**

This review outlines all published biomarkers for ICI therapy and highlights potential candidate markers for future research. To date, PD-L1 is the best studied biomarker for PD-1 ICI response. The most promising candidate predictive biomarkers for ICI response have not yet been identified. Variations in outcome parameters, statistical power, and analyses hampered summary of the results. Further investigation of biomarkers in larger patient cohorts using standardized objectives and outcome measures is recommended.

## Introduction

### Rationale

Immune checkpoint inhibitors (ICIs) represent a major breakthrough in treatment of metastatic melanoma and are currently also investigated in other types of cancer. These antibodies target CTLA-4 or PD-1 molecules on T-cells, resulting in prolonged activation of T-cell responses, including potential tumor-reactive T-cell responses. Impressive long-term survival up to 5 years has been seen in advanced melanoma patients upon treatment with ICI (1, 2), indicating that activation of the immune system can be effective in inhibiting cancer progression in patients. However, despite the promising results with ICI, response rates of advanced melanoma patients are still low or moderate. Less than 20% of advanced melanoma patients experience a long-term response to ipilimumab (2). PD-1 ICI has been proven effective in a larger set of patients, but durable responses to these therapies are limited to 30–40% of patients (3), or up to 60% for a combination of these drugs (4). This means that durable responses are still not seen in over 40% of ICI-treated patients.

Moreover, treatment with ICI therapies can confer severe and potentially life-threatening side-effects, such as diarrhea, enterocolitis, hepatitis, hypophysitis, skin rash, and pruritus. These immune-related adverse events (IRAEs) were seen in up to 80% of patients in clinical trials with ipilimumab, of which 10–17% was reported to be grade 3 or higher. Consequently, ipilimumab-treated patients frequently suffer from toxicities, while only 20% is expected to benefit from treatment. These figures call for predictive biomarkers for ICI therapy responsiveness of advanced melanoma patients (5). Biomarkers predicting treatment response of ICI in metastatic melanoma will be instrumental to (1) enable personalized treatment with ICI selecting those patients with likely benefit from ICI, while other patients can proceed to other therapies, without treatment delay due to unresponsiveness to ICI, (2) avoid suffering of potentially severe adverse effects by patients who are not likely to have clinical benefit, and (3) increase cost effectiveness.

Several classes of immunological correlates have been associated with the administration of ICI, indicating the potential usefulness of correlates as predictive or prognostic markers for response, survival, and IRAEs. Predictive markers would have a significant impact on clinical decision making in choosing ICI and enable medical treatment to be tailored accordingly.

### Objectives

Here, we systematically review the current literature of clinical data of ICI treatment to provide an overview of candidate predictive biomarkers for ICI in melanoma patients.

### Research Question

Which candidate predictive biomarkers for ICIs have been studied in melanoma patients?

## Methods

### Study Design and Search Strategy

A medical information specialist (Jacqueline Limpens) performed a systematic search in OVID MEDLINE from January 1, 2000 to August 15, 2016 to identify publications in English on biomarkers predicting the clinical response to ICI treatment of human melanoma. We checked the availability of systematic reviews on this topic and only included original articles in our review. Original articles were found by safely excluding reviews and editorials by double negation (i.e., excluding “reviews” as publication type, except when terms indicating observational studies or trials were present; the same approach is used to safely exclude animal studies). To find both known and unknown biomarkers, the concepts melanoma and ICI were combined with either (I) general terms for biomarkers or predictive factors or (II) known specific factors combined with terms for prognosis, correlation, predictors and terms for survival, mortality, and clinical response. (See Table S1 in Supplementary Material for entire MEDLINE search strategy.) In addition, forward and backward snowballing was performed of identified relevant publications.

### Participants, Interventions, and Comparators

Multiple study designs were considered for this review, including clinical trials (phase I, II, and III), prospective or retrospective cohort studies, and case series that reported on clinical response and/or survival outcomes to ICI therapy. Case reports and other type of publications including reviews, viewpoints, or conference reports were excluded. All original research publications in English were included. The patient population of this review comprised AJCC stage 3 or 4 melanoma patients who are eligible to receive ICI therapy. Inclusion eligibility required publications to report on melanoma patients treated with either anti-CTLA-4 antibodies (ipilimumab, tremelimumab, and ticilimumab) or anti-PD1 antibodies (nivolumab and pembrolizumab). Further eligibility required biomarker analysis at baseline prior to treatment. No restrictions were made on the type of outcome parameter for response or survival. No restrictions regarding age, sex, or ethnicity were applied. Publications were excluded when reporting on (1) non-melanoma patients, (2) exclusively biomarkers during treatment, (3) animal studies, (4) exclusively biomarkers for IRAEs, and (5) combination therapy of ICI with other melanoma drugs (except peptide vaccinations).

### Systematic Review Protocol and Data Extraction

Selection of publications and data extraction was performed independently and in an unblinded standardized manner by two reviewers (Charissa A. C. Jessurun and Julien A. M. Vos). Any disagreement between reviewers was resolved by consensus. The following information was extracted from selected publications: study type, type of ICI therapy, number of patients included in the study, number of patients included in the biomarker analysis, type of predictive biomarker, outcome parameter, clinical outcome measure, and statistical significance. The outcome parameters of the studies were divided in three groups: (1) clinical response, (2) progression-free survival (PFS), and (3) overall survival (OS). PFS also included disease-free interval, response duration, relapse-free survival, and recurrence-free survival. Clinical response also comprised tumor response, best overall response rate, immune-related response criteria, and clinical benefit.

### Data Analysis

This review was setup to generate an inventory of published candidate biomarkers. Statistical analysis was not feasible due to various outcome measures and limited number of studies per type of biomarker. Therefore, to provide preliminary insights, the predictive values of biomarkers were extracted per publications and summarized as the number of biomarkers studies reporting significance at a level of 0.05.

### Quality Assessment

A risk of bias analysis was performed on all included publications based on the Cochrane Collaboration quality checklist for prognostic studies, consisting of the following eight questions: (1) Are the participants adequately described and are the reasons for any restrictions appropriate? (2) Are the biomarkers specified? (3) Are the measurements of assessment valid and reliable? (4) Is the follow-up data clearly described? (5) Is there sufficient data on biomarkers in the study population? (6) Type of prognostic study: one biomarker or multiple biomarkers? (7) Phase of the study: test population, validation population, impact study? and (8) Type of outcome: point estimate, survival curve, prognostic model, or impact study. Questions 1–5 were answered with yes, no, or questionable. Questions 6–8 were not used for the risk of bias assessment. Publications with at least four times “yes” in questions 1–5 were assessed as low risk of bias. Publications scoring 1 “questionable” in either question 4 or question 5 or 2 “questionable” in questions 1–5 were assessed as intermediate risk of bias. High risk of bias was assigned to publications scoring 2 “questionable” in question 4 and 5 or 1 “no” in question 4 and/or 5. Publications describing the analyses of multiple types of biomarkers were assessed for the quality of the analysis of each biomarker type separately.

## Results

### Study Selection and Characteristics

**Figure 1:**
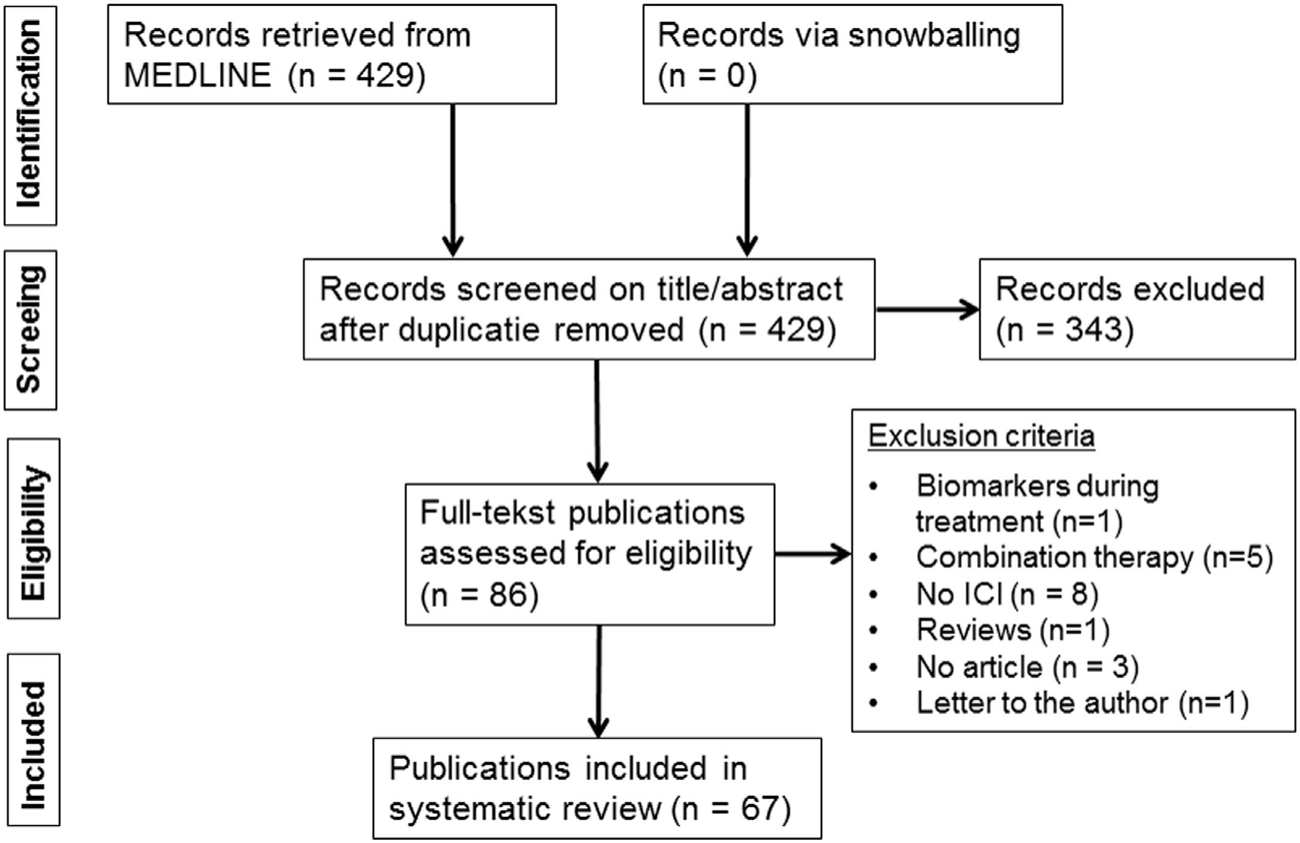
Flow diagram of included publications.

The systematic MEDLINE search retrieved 429 publications (Figure 1). Snowballing did not yield extra publications. Based on the eligibility criteria of title and abstract screening, 343 publications were excluded and 86 publications were screened full text, of which 67 publications met our selection criteria and were included in this review (6–72) (Table S2 in Supplementary Material). The study selection process and reasons for exclusion are presented in Figure 1.

**Figure 2:**
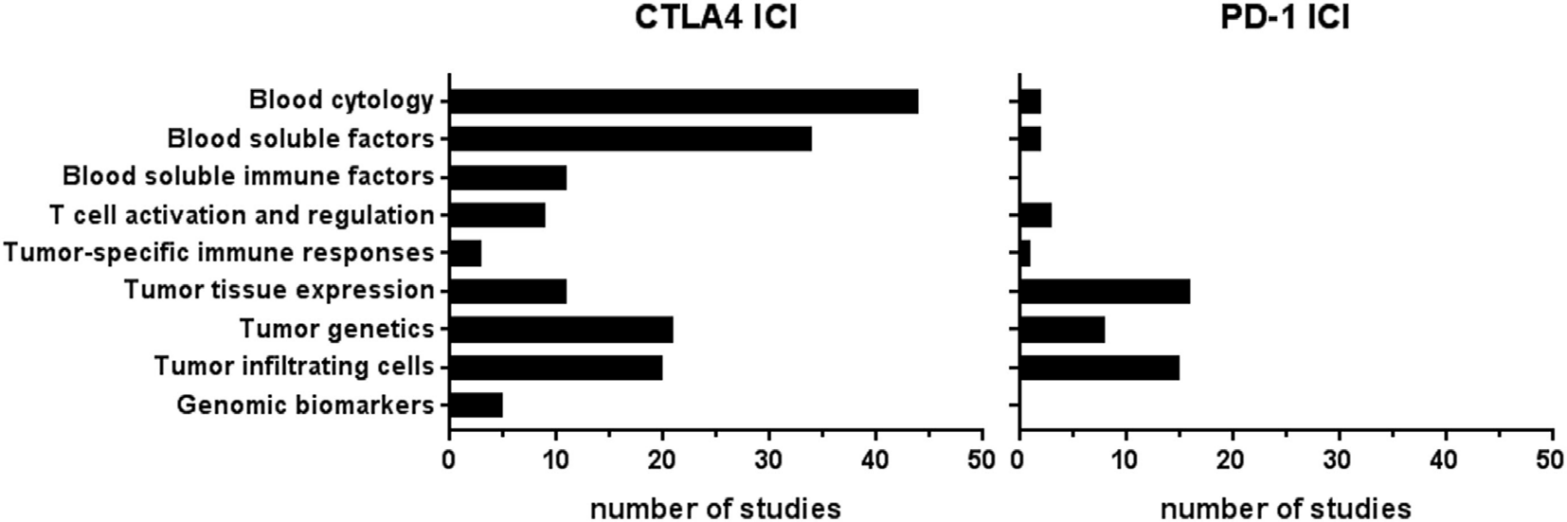
Number of studies per biomarker type.

In total, 67 publications reported on 57 types of biomarkers (Table S2 in Supplementary Material). Of these 67 publications, 54 publications reported on more than one biomarker, resulting in 205 biomarker studies. Most biomarker studies were based on CTLA-4 ICI therapy (*n* = 158), whereas 47 studies were performed for PD-1 biomarkers. The types of biomarkers were divided into three groups, depending on the tissue type: (1) blood, (2) tumor tissue, and (3) genomic DNA from normal tissue. The blood-based biomarker group included studies on general cytology markers, general soluble factors, immune-related soluble factors, cellular markers of T-cell activation and regulation, and systemic tumor-specific immune responses (Figure 2). These biomarkers were reported in 101 studies relating to anti-CTLA-4 and in 8 studies relating to anti-PD1 therapy. The second group, focusing on tumor tissue-based markers such as expression profiles, genetic alterations, and tumor-infiltrating cells, was described in 52 studies for anti-CTLA-4 and 39 for anti-PD1. This indicates a predominant interest in these markers for PD-1 ICI. The third group comprised markers based on genomic DNA of normal tissue and included five studies for CTLA-4 ICI and none for PD-1 ICI. The median number of patients included in the biomarker studies varied from 69 and 65 patients for blood and genomic biomarker studies, respectively, to 28 patients in tumor tissue studies (Table 1).

**Table 1:** Study population size per biomarker category.

Type of biomarkers studied at baseline	No. patients per analysis^a^	IQR
Blood biomarkers (total)	69	(138–125)
Blood cytology	79	(59–138)
Blood soluble factors	95	(51–128)
Blood soluble immune factors	40	(35–247)
T cell activation/regulation	54	(12–109)
Tumor-specific immune responses	22	(22–48)
Tumor tissue biomarkers (total)	28	(16–39)
Tumor tissue expression	16	(16–28)
Tumor genetics	38	(38–76)
Tumor-infiltrating cells	24	(13–37)
Genomic biomarkers	65	(40–67)

*^a^Median number of patients per analysis*.

*IQR, interquartile range*.

### Risk of Bias

**Figure 3:**
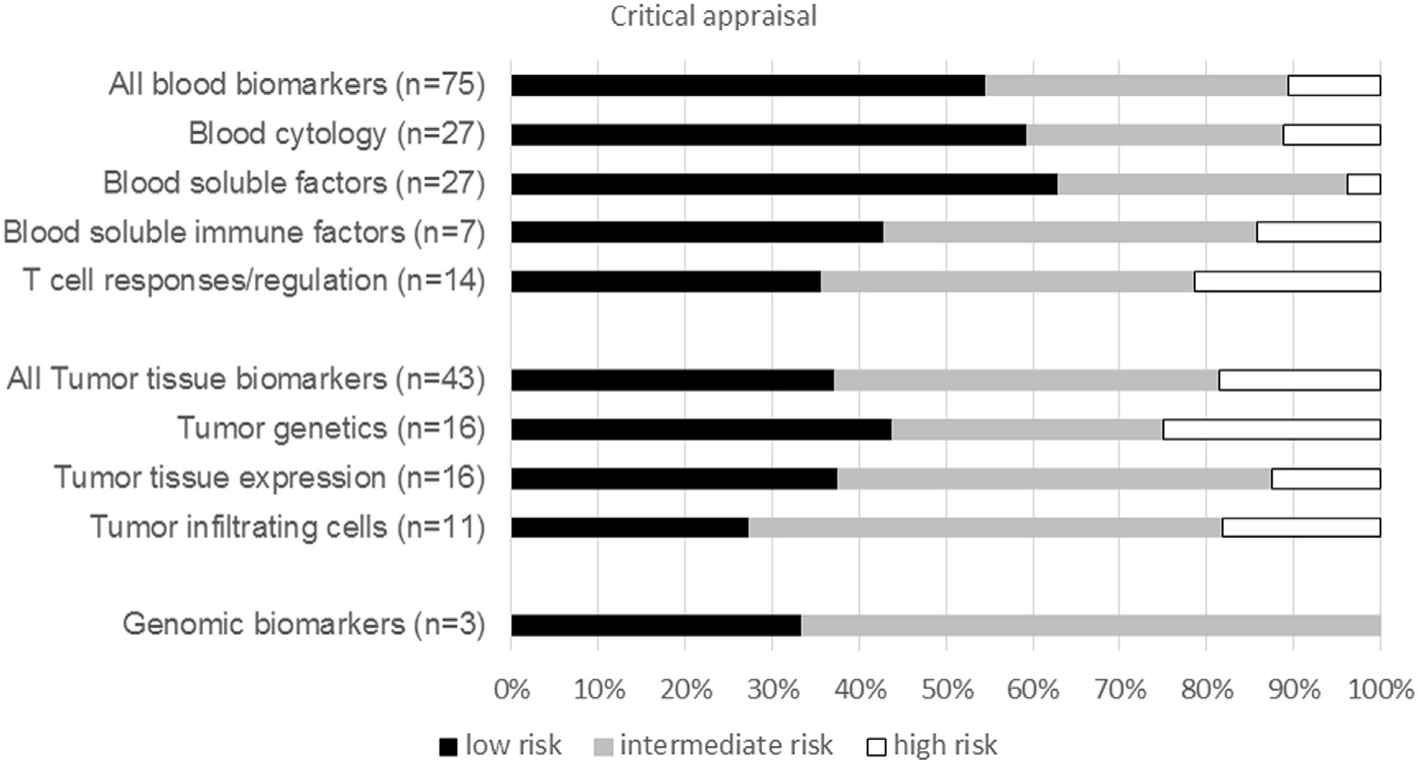
Critical appraisal. Graph show the percentage of publications per biomarker type for both CTLA-4 and PD1 immune checkpoint inhibitor with a low, intermediate, or high risk of bias. *n*, total number of publications per biomarker type.

The risk of bias was assessed for each biomarker type including both CTLA-4 and PD-1 ICI publications (Figure 3). The assessment revealed a low risk of bias in 44% of all publications, intermediate risk in 42%, and high risk of bias in 14% of publications. Publications describing blood biomarkers had less risk of bias than tumor tissue or genomic biomarker publications. Within blood biomarker publications, the highest risk of bias was estimated in the T-cell activation and regulation subgroup, whereas the lowest risk of bias was found in the blood soluble factor subgroup. The publications of tumor tissue biomarker types were comparable in risk of bias, except for a lower risk of bias in the tumor genetic subgroup (Figure 3). The risk of bias was mostly due to an unclear description of follow-up data of patients or insufficient data on the biomarker in the study population. The development phase of the biomarker publications was predominantly test population-based (*n* = 60) and seven publications consisted of both test population and validation studies. No publications describing impact studies were retrieved.

### Blood Biomarkers

**Figure 4:**
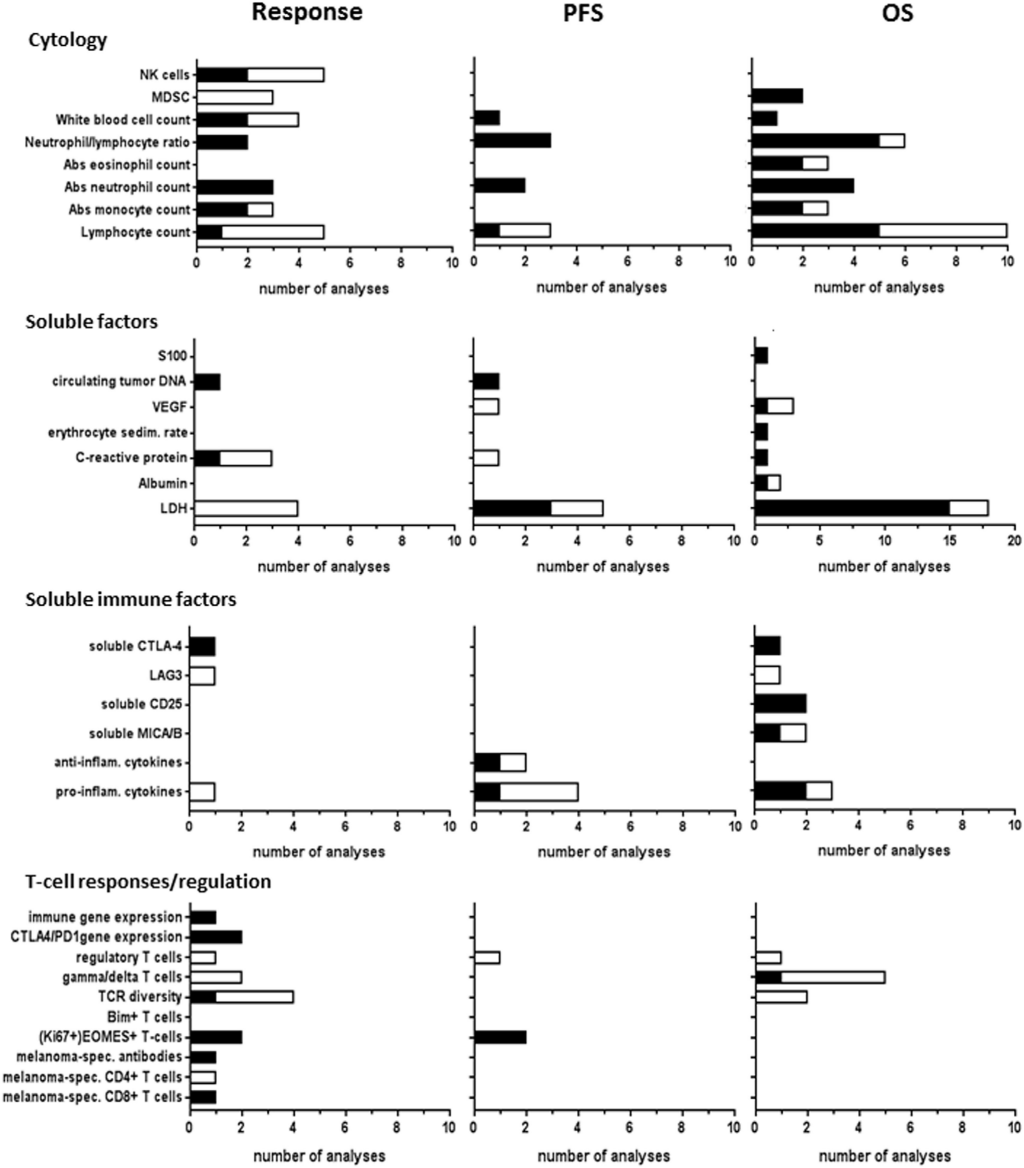
Blood biomarkers analyzed for CTLA-4 immune checkpoint inhibitor (ICI). Graphs show the number of analyses per biomarker type for correlations with clinical response, progression-free survival (PFS), or overall survival (OS) upon CTLA-4 ICI therapy. Black bars, significant correlation; white bars, non-significant correlation.

Blood cytology biomarkers studies included baseline absolute counts of white blood cells, lymphocytes, granulocytes (eosinophils or neutrophils), monocytes, myeloid-derived suppressor cells (MDSCs), natural killer (NK) cells, as well as ratios between neutrophils and lymphocytes (Figure 4). These blood biomarkers were most frequently analyzed for correlations with clinical response and OS to CTLA-4 ICI therapy. Correlations to response were found in approximately half of the analyses, whereas correlations to OS were found in the majority of analyses. MDSC analyses revealed significant correlations to OS, but not to response. In PD-1 ICI-treated patients, MDSCs were the only blood cytology marker reporting significant correlations to all outcome parameters (23, 66).

Soluble blood factors studied in CTLA-4 ICI-treated patients included S100, circulating tumor DNA, vascular endothelial growth factor, erythrocyte sedimentation rate, C-reactive protein, albumin, and lactate dehydrogenase (LDH). LDH was most frequently studied and showed significant correlations to OS and PFS, whereas no correlations to response were found. LDH also correlated to OS in one PD-1 ICI study (17). One study on circulating tumor DNA reported significant correlations to both response and PFS for both CTLA-4 and PD-1 ICI (24).

Immune-related soluble factors in the blood were reported in 11 CTLA-4 ICI studies and in none of the PD-1 ICI studies. These factors included pro-inflammatory cytokines, anti-inflammatory cytokines, soluble MIC A or MICB, CD25, LAG3, and soluble CTLA-4 (Figure 4). Four of these markers showed significant correlations to OS. Soluble CTLA-4 correlated to both response and OS (40), whereas soluble LAG3 did not (13, 26).

It is conceivable that to trigger an immune response against a tumor using ICI, a patient should display an active immune microenvironment. In 12 CTLA-4 ICI studies, biomarkers for systemic T-cell activation and regulation in the blood were mostly analyzed as response markers, comprising eomesodermin (EOMES)-positive CD8+ T-cells, Bim+ T-cells, T-cell receptor (TCR) diversity, γδ T-cell counts, regulatory T-cell frequency, and expression of CTLA-4, PD-1, or immune-related genes (Figure 4). EOMES+ T-cells significantly correlated to both response and PFS in two analyses of either total EOMES+ T-cells or the Ki67+ proliferating fraction of these cells (65). Baseline regulatory T-cell frequencies neither correlated to any outcome in CTLA-4 studies (42, 47, 56) nor to PFS upon PD-1 ICI therapy (23). Bim-expressing T-cell levels (18) correlated to PD-1 ICI response. Both TCR diversity (62) and Bim-expressing T-cell levels (18) correlated to PD-1 ICI response. The presence of melanoma antigen-specific T-cell responses at baseline can greatly enhance the response rate of ICI, by amplifying pre-existing T-cell responses (58). High baseline TCR diversity has been associated with ICI response in both anti-CTLA4 and anti-PD1 (45, 62). However, no relevant differences in response to tremelimumab were seen in the small study by Robert et al. (50). Baseline tumor-specific CD8+ T-cell responses showed correlations to both CTLA-4 ICI (70) and PD-1 ICI (67) response, but potential correlations to PFS or OS were not analyzed. No correlation of baseline melanoma-specific CD4+ T-cell immunity to clinical response was found for CTLA-4 ICI (70). Pre-existing melanoma antigen-specific antibody response was only studied for CTLA-4 ICI (70) showing a correlation to response. In general, melanoma-specific immunity was more extensively studied during therapy for therapeutic response monitoring, not included in this review, rather than as predictive biomarker.

### Tumor Tissue Biomarkers

**Figure 5:**
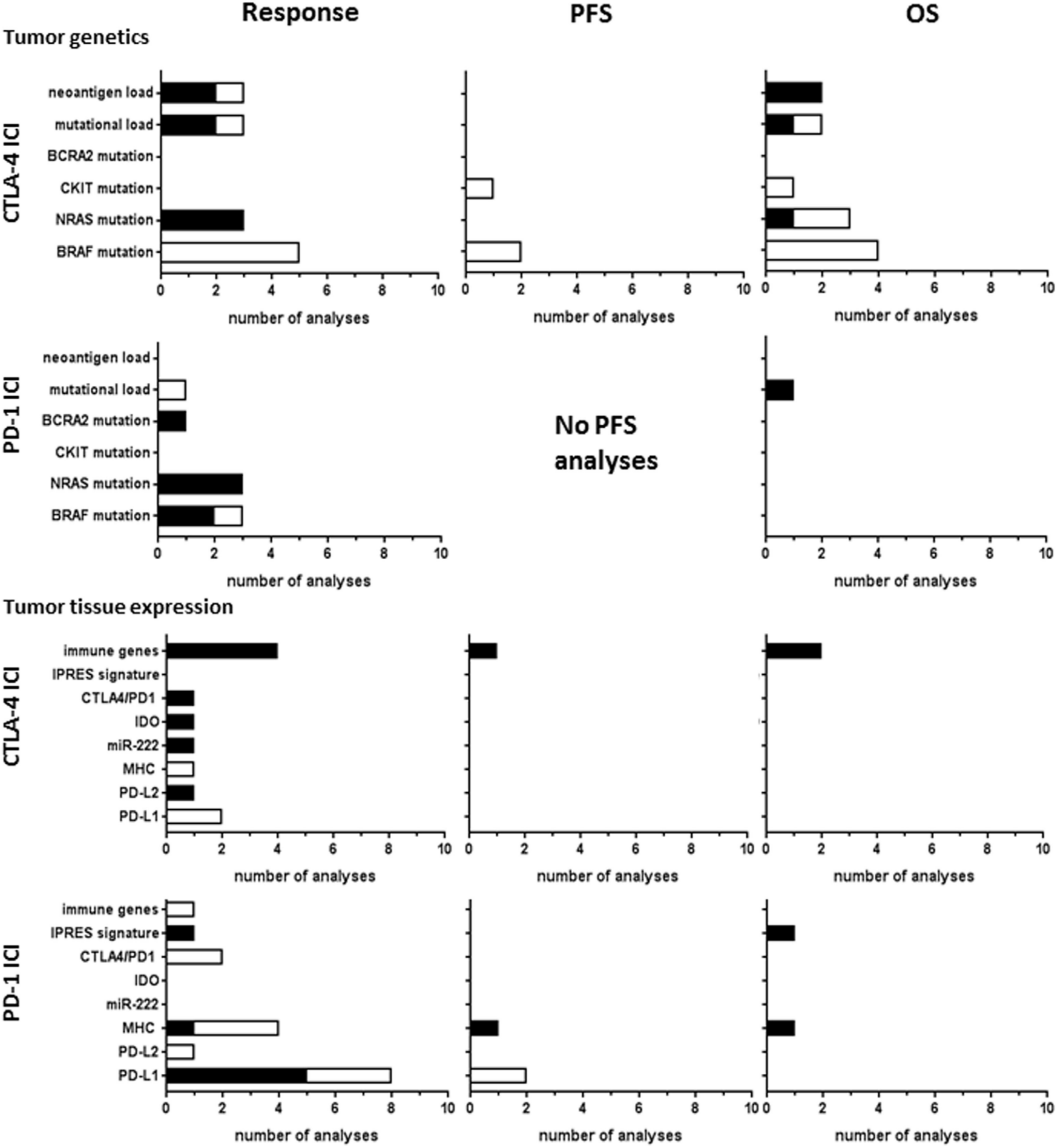
Tumor tissue biomarkers analyzed for CTLA-4 or PD-1 immune checkpoint inhibitor (ICI). Graphs show the number of analyses per biomarker type for correlations with clinical response, progression-free survival (PFS), or overall survival (OS) upon CTLA-4 ICI therapy. Black bars, significant correlation; white bars, non-significant correlation.

Predictive biomarkers based on tumor-intrinsic factors or the tumor microenvironment have been investigated for both CTLA-4 and PD1 ICI. Known oncogenic mutations were studied in 15 CTLA-4 ICI studies and 7 PD-1 ICI studies (Figure 5). BRAF V600E mutations did not correlate to response or OS in any of the nine studies of CTLA-4 ICI, whereas two out of three studies reported a significant correlation to PD-1 ICI response. N-RAS mutations correlated to response in three studies of CTLA-4 and PD-1 ICI, while PFS and OS were hardly analyzed. In a large genetic screening, BRCA2 mutations were more frequently found in responding tumors to PD-1 ICI than in non-responding tumors (27).

Mutational load of the tumor is thought to influence the clinical benefit from ICI by generating tumor-specific neoantigens derived from tumor mutations that mediate T-cell responses against the tumor (73). The study by Hugo et al. investigated the genomic and transcriptomic features of tumors responding to PD-1 ICI (27). They reported significant correlations of a high mutational load in tumor tissue to OS, whereas the statistical significance cutoff was not met for clinical response (27). Mutational load was analyzed in three studies of CTLA-4 ICI. Snyder et al. (57) found significant correlations to response and OS and Van Allen et al. (64) reported a correlation of non-synonymous mutational load to clinical response. This association was not found in another study analyzing the mutational load in clinically used cancer gene panels, composed of ~300–600 well-characterized cancer-related genes (12). These gene panels can therefore not be used as simplified approach to estimating the mutational load for biomarker application.

The presence of neoantigens derived from tumor mutations was investigated in three studies of CTLA-4 ICI. Snyder et al. identified shared tetrapeptide neoepitopes encoded by mutations in diverse genes across the genome, which correlated to ICI response (57). Likewise, Van Allen et al. reported a significant correlation of neoantigen load to response (64). However, in contrast to Snyder et al., the neoantigens found in these patients were almost all unique antigens, as only 0.04% of them were recurrent antigens in multiple patients. The same investigators contributed to the latest study of McGranahan et al., which reports improved OS in patients exhibiting low neoantigen intratumor heterogeneity and high clonal neoantigen burden (43). This indicates that the correlation of neoantigen load with OS becomes more significant when combined with neoantigen intratumor heterogeneity.

Tumor tissue expression biomarkers were addressed in 11 CTLA-4 ICI studies and 16 PD1 ICI studies (Figure 5). Expression of the main PD-1 ligand PD-L1 showed significant correlations to response in five out of eight analyses, whereas no significant correlation to PFS was found. PD-L1 did not correlate to CTLA-4 ICI response (13, 54). The other PD-1 ligand, PD-L2, was less frequently expressed in tumor tissues studied. Nevertheless, increased PD-L2 expression in tumors of patients responding to CTLA-4 ICI therapy was found (64), which was not found for PD-1 ICI therapy (60). Studies of T-cell checkpoint gene expression by RNA analyses found a significant correlation of CTLA-4 expression with response to CTLA-4 ICI (64), but not to PD-1 ICI (27). Higher baseline expression of indoleamine 2,3-dioxygenase (IDO) was positively associated with clinical response to CTLA-4 ICI in one study (25). Although IDO has been associated with an immunosuppressive tumor microenvironment, the authors argued that its upregulation may also result from an ongoing, albeit suboptimal, antitumor immune response, as explanation for the observed positive association with response. The study by Galore-Haskel et al. reported an immune resistance mechanism of melanoma that is controlled by the RNA editing protein ADAR1 (21). ADAR1 regulates the synthesis of miR-222, which controls ICAM1 expression and affects immune resistance. In their study, miR-222 was significantly more expressed in tumors of patients responding to ipilimumab and therefore suggested as candidate biomarker (21).

The expression of immune-related genes, also referred to as immune gene signature, of tumor tissues correlated to CTLA-4 ICI response in all four studies, two of which also found correlations to PFS or OS. For PD-1 ICI therapy, Hugo et al. found that PD-1 ICI resistant tumors display a transcriptional signature, referred to as innate anti-PD1 resistance (27), characterized by expression of genes involved in mesenchymal transition, cell adhesion, ECM remodeling, angiogenesis, and wound healing.

**Figure 6:**
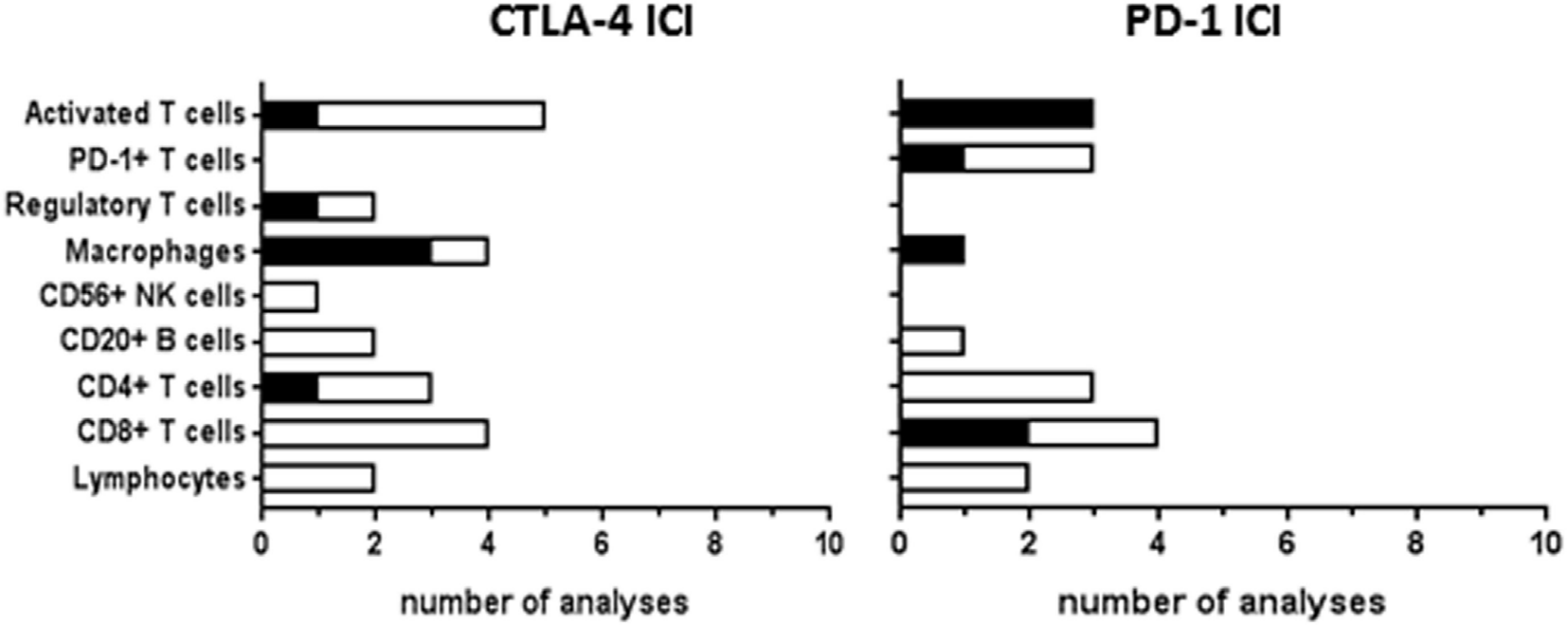
Tumor-infiltrating cells analyzed as immune checkpoint inhibitor (ICI) response biomarker. Graphs show the number of analyses per biomarker type for correlations with clinical response to CTLA-4 or PD-1 ICI therapy. Black bars, significant correlation; white bars, non-significant correlation. Progression-free survival and overall survival were not analyzed in most of the studies.

Tumor-infiltrating cells at baseline were investigated for response correlations in 20 CTLA-4 ICI studies and 15 PD-1 ICI studies (Figure 6). The studies of immune-related gene expression in tumor tissue, as described earlier, also included genes involved in T-cell activation and cytolytic activity, such as granzyme B or perforin (25, 64). Gene expression correlated to clinical response in the study by Van Allen et al. (64), which was not found in the earlier study of Hamid et al. (25). Immunohistochemical analyses of baseline total lymphocytic infiltrate did not show any correlation to CTLA-4 or PD-1 ICI response. Tumor infiltration of CD8+ T-cells was analyzed more frequently, showing correlations to response in two out of four PD-1 analyses but in none of the four CTLA-4 ICI analyses. Tumeh et al. proposed a predictive model for PD-1 ICI response based on the invasive margin CD8+ T-cell density, which was validated in a separate patient cohort, indicating that the location of CD8+ T-cell infiltration within the tumor is of clinical importance (62). Intratumoral memory T-cell density correlated to PD-1 ICI response (13), which was not found for CTLA-4 ICI (25). Baseline infiltration of CD4+ T-cells, B cells, or NK cells hardly showed any significant correlations nor did baseline levels of regulatory T-cells. However, correlations were found of intratumoral macrophage density, their proximity to CD8+ T cells, or the ratio between CD68+ and CD163+ phenotype with clinical response to CTLA-4 ICI or to PD-1 ICI (13, 51). Similar to the systemic antitumor T-cells responses, tumor-infiltrating cells were mostly studied during therapy to monitor the induction of antitumor immunity by ICI therapy (not included in this review).

### Genomic Biomarkers

Genomic biomarkers on normal tissue (e.g., blood) were studied for their predictive value for CTLA-4 ICI, including polymorphisms in the CTLA-4 gene and in other genes involved in immune activation and the presence HLA-A alleles. No genomic biomarker studies for PD-1 ICI were retrieved. Of 14 single-nucleotide polymorphisms (SNPs) tested in the CTLA-4 gene, 4 SNPs showed significant correlations to clinical response (11, 25, 46). Queirolo et al. found correlations of two out of six CTLA-4 SNPs to OS, as well as significant correlations with combined haplotypes of these two SNPs, in a relatively small cohort of 14 patients, suggesting a strong association (46). Hamid et al. performed genotyping of 20 SNPs and 2 deletions in 10 immune-related genes (BTNL2, CCR5, CD86, CTLA-4, IFNAR1, IFNAR2, IFNG, IL23R, NOD2, and PTPN22) (25), but found no significant correlations to clinical response probably due to insufficient statistical power. Moreover, no associations were found between the presence of the common HLA-A alleles, including HLA-A*0201, and clinical response.

## Discussion

### Summary of the Main Findings

This review provides a systematic overview of candidate predictive biomarkers and their correlations to clinical outcome parameters for ICI treatment in melanoma patients. In addition to predictive markers, part of the included publications also described biomarkers during therapy, not assessed in this review. Three types of baseline biomarkers were discriminated based on the tissue type: blood, tumor tissue, and genomic DNA of normal tissue. Biomarkers studies of CTLA-4 ICI addressed all biomarker types, whereas PD-1 ICI studies mainly addressed tumor tissue biomarkers. This shows a predominant interest in tumor tissue biomarkers for PD-1 ICI. The majority of biomarkers studies were of exploratory nature. Correlation analyses were focused predominantly on response and OS, while PFS correlations were less frequently reported.

Blood cytology markers and markers associated with systemic T-cell responses and regulation correlated to clinical response in CTLA-4 ICI studies. However, cytology markers also correlated to OS, which may indicate their prognostic, independent of the therapy given, rather than predictive value. Especially, the frequent correlation of LDH to OS, but not to response, confirms the prognostic character of LDH. Baseline MDSC levels showed a similar prognostic pattern. As several biomarkers were only analyzed for associations with survival outcomes (PFS and OS), their predictive potential for therapy response remains unclear.

Tumor tissue biomarkers were predominantly analyzed for correlations to response for both CTLA-4 and PD-1 ICI. Tumor mutations and neoantigen load, as well as the expression of immune-related genes in tumor tissue and/or the presence of CD8+ T-cell infiltrate, showed significant correlations to response in large genetic and transcriptional analyses of tumor tissue. This indicates the importance of tumor antigen levels, recognized by activated tumor-infiltrating T-cells, for the response rate of ICI therapy. Further research will be necessary to evaluate the power and applicability of these biomarkers for predictive tests. To date, PD-L1 is the best studied biomarker for PD-1 ICI response. Although the results vary, the predictive potential of PD-L1 was confirmed in a recent meta-analysis of published and unpublished data presented at conferences (74).

### Limitations

A potential limitation of tumor tissue biomarkers might be that they were assessed on excised tissue prior to therapy, which does not reveal the variation in tumor tissue phenotype at other (metastatic) sites or expression variations in time. Tumeh et al. showed that intratumoral PD-L1 expression is associated with pre-existing CD8+ T-cell infiltration (62). Since PD-L1 is upregulated by interferons produced by tumor-infiltrating T-cells and subsequently blocks T-cell activation in a negative feedback loop, the predictive value of PD-L1 may dependent on the presence of intratumoral T-cell infiltration. The tumor infiltrate studies also indicated an association of macrophages with response to both ICI types, whereas the other cell types yielded varying results. These varying results may have resulted from differences in detection methods used for T-cells and their activation status, indicating another limitation to summarizing these biomarker studies.

Genomic analyses revealed polymorphisms in the CTLA-4 gene as predictive biomarker for CTLA-4 ICI. However, genomic biomarker studies, including polymorphisms in immune-related genes, were the least studied biomarker type and deserve further investigation in larger study populations for both ICI to reveal their predictive value.

The interpretation and summary of biomarker results among studies remained difficult due to diversity in outcomes parameters used, in particular the definition of response to ICI. ICI response can be measured according to tumor response criteria or as survival prolongation. Variations in the cutoff value of these parameters, to dichotomize responding and non-responding patients, can greatly affect the results. Moreover, as survival is influenced by many confounding factors, survival associations are less informative for ICI response prediction. Biomarker studies reported variable significance, probably due to limited numbers of patient and low statistical power. The analyses also differed in level of statistics. Some publications only reported univariate analyses, whereas others also included confounding factors in multivariate analyses. For future biomarkers research, it is therefore recommended to use well-defined patient populations and standardized outcome measures to enable meta-analysis of the biomarker results.

## Conclusion

The most promising candidate predictive biomarkers for ICI response have not yet been identified. To date, PD-L1 is the best studied biomarker for PD-1 ICI response. This review outlines all published biomarkers for ICI therapy and highlights potential candidate markers for future research. In addition to investigating candidate biomarkers, further understanding of the mechanism of action of ICI therapy will support the identification of new predictive biomarkers.

## Author Contributions

CJ and JV contributed equally. All authors were involved in the design of the study, organization of the study, manuscript writing, and approval of final version of the manuscript. JL, CJ, and JV generated the search strategy and performed the search. CJ and JV independently performed the article selection procedure, data extraction, and summarized the data. RL checked the data analysis and validity and composed the figures. CJ, JV, and RL wrote the manuscript. JL gave advice to the manuscript text.

## Conflict of Interest Statement

The authors declare that the research was conducted in the absence of any commercial or financial relationships that could be construed as a potential conflict of interest.
