# Supplementary material for: Biomarkers for Response of Melanoma Patients to Immune Checkpoint Inhibitors: A Systematic Review
Source: Front Oncol. 2017 Sep 27;7:233. doi: 10.3389/fonc.2017.00233 (PMC5625582; doi:10.3389/fonc.2017.00233)
Supplement: Supplementary file 1 [file Data_Sheet_1.docx]

**Supplementary Table S1:** **MEDLINE search**

**Database(s): Epub Ahead of Print, In-Process & Other Non-Indexed Citations, Ovid MEDLINE(R) Daily and Ovid MEDLINE(R)** 1946 to Present **Search Strategy: 2016-08-15**

| **#** | **Searches** | **Results** |
| --- | --- | --- |
| 1 | "melanoma"/ | 73809 |
| 2 | melanom*.tw,kf,ot,jw. | 97898 |
| 3 | 1 or 2 [melanoma] | 110527 |
| 4 | ((exp animals/ or animal experimentation/ or disease models, animal/ or animal diseases/ or exp animals, genetically modified/ or (mice or mouse or murine or animal*).ti. or ((mouse or murine or animal or trangenic) adj3 (model or melanom*)).tw.) not (humans/ or (human* or patient*).ti.)) or (rodent* or squirrel* or marmoset* or rat or rats or rabbit* or sheep or lamb or lambs or ewe or ewes or ovine or goat* or pig or pigs or piglet* or porcine or sus or swine* or sow or sows or gilt or gilts or dog or dogs or bitch or bitches or canine or cat or cats or feline or primate* or monkey* or macac* or macaq* or rhesus).ti. or (dam or dams or pup or pups).tw,kf. | 4529903 |
| **5** | **3 not 4 [human melanoma]** | **97478** |
| **6** | **limit 5 to yr="2000 -Current" [human melanoma > 2000**] | **57602** |
| **7** | **limit 6 to (dutch or english)** [ Dutch and English studies on human melanoma > 2000] | **54101** |
| 8 | ctla-4 antigen/ai or programmed cell death 1 receptor/ai | 655 |
| 9 | (ipilimumab or nivolumab or pembrolizumab or tremelimumab).tw,kf,ot,rn. | 2247 |
| 10 | (MDX-CTLA-4 or MDX-CTLA4 or Yervoy or MDX010 or MDX-010 or MDX101 or MDX-101 or MDX1106 or MDX-1106 or ONO4538 or ONO-4538 or BMS936558 or BMS-936558 or Opdivo or lambrolizumab or Keytruda or MK3475 or MK-3475 or SCH900475 or SCH-900475 or ticilimumab or CP-675* or CP675*).tw,kf,ot. | 181 |
| 11 | ((CTLA-4* or CTLA4* or cytotoxic-T-lymphocyte-associated-4 or cytotoxic T-lymphocyte-antigen-4 or cytotoxic-T lymphocyte-associated antigen-4 or CTL-antigen*-4* or CTL-associated-antigen*-4* or CD152 or CD-152 or CD279* or CD-279* or PD-1* or PD1* or PDL1* or PD-L1* or PDL-1* or checkpoint* or CHK* or programmed death or programmed cell death) adj6 (inhibitor* or block* or antagon* or therap* or immunother* or vaccin* or antibody treatm* or targeting)).tw,kf. | 10791 |
| 12 | (anti adj (CTLA-4* or CTLA4* or cytotoxic-T-lymphocyte-associated-4 or cytotoxic T-lymphocyte-antigen-4 or cytotoxic-T lymphocyte-associated antigen-4 or CTL-antigen*-4* or CTL-associated-antigen*-4* or CD152 or CD-152 or CD279* or CD-279* or PD-1* or PD1* or PDL1* or PD-L1* or PDL-1* or checkpoint* or CHK* or programmed death or programmed cell death)).tw,kf. | 1749 |
| **13** | **or/8-12 [immune checkpoint inhibitors]** | **12379** |
| **14** | **7 and 13 [human melanoma + immune checkpoint inhibitors]** | **2048** |
| 15 | meta-analysis/ or (meta analy* or metaanaly* or meta?analy*).tw,kf. or ((systematic* adj3 (review or literature or evidence or search*)) or ((summari* or review) adj3 evidence) or (search* adj12 (literature* or ((electronic or medical or biomedical) adj3 database*) or exhaustive or comprehensive or medline or pubmed or embase or psychinfo or (CENTRAL and cochrane) or "Central Register of Controlled Trials"))).tw. or (cochrane or clinical evidence or EBM).jw. [secondary study-filter] | 263646 |
| **16** | **14 and 15 [secondary studies on melanoma + immune checkpoint inhibitors]** | **85** |
| 17 | editorial/ or books/ or (expert or current or cochrane or clinical evidence or EBM).jw. or (systematic* adj3 (review or literature)).ti. or ((review/ or (systematic* adj3 (review or literature or search*)).tw,kf. or (search* adj12 (literature* or ((electronic or medical or biomedical) adj3 database*) or exhaustive or systematic or medline or pubmed or embase or psychinfo or (CENTRAL and cochrane) or "Central Register of Controlled Trials")).tw.) not (clinical trial/ or exp controlled clinical trial/ or multicenter study/ or observational study/ or comparative study/ or exp cohort studies/ or case-control studies/ or exp databases, factual/ or datasets as topic/ or exp population surveillance/ or ("own study" or "own data" or "our data" or retrospective study or prospective study or follow-up study or case control study or cohort or observational study or hospital based or population-based or study-population or consecutive or (cumulative adj3 (incidenc* or probabil*)) or registry* or registries or ((register or registers) not (Cochrane adj3 register*)) or nationwide or nation-wide or community-wide or real-life or real-world or ((national or international) adj3 (data or databas*)) or long-term trend* or (contempor* adj3 (setting* or rate* or pattern* or "use" or practice* or populat* or data))).tw. or medical record*.hw. or ((review adj2 (case or cases)) or case serie*).tw.)) [filter to exclude reviews and editorials] | 2559097 |
| **18** | **14 not 17 [primary studies on melanoma + immune checkpoint inhibitors]** | **1352** |
| 19 | models, biological/ or biological factors/ or biomarkers/ or biomarkers, pharmacological/ or biomarkers, tumor/ or genetic markers/ | 657163 |
| 20 | multivariate analysis/ | 101806 |
| 21 | (biomark* or marker* or surrogate* or predictor* or indicator* or selection criter* or multivariate or univariate).tw,kf. | 1389714 |
| 22 | (predict* or prognos*).ti. | 343910 |
| 23 | ((prognos* or predict*) adj4 (factor* or value* or abilit* or accura* or scor* or parameter* or variable* or significance or relevance or role*1 or impact or respons* or benefit* or positive or negative or model* or signature or validat*)).tw,kf. | 542850 |
| 24 | ((factor* or feature or features or parameter* or variables or signature) adj4 (associat* or biologic* or biochem* or molecular* or genet* or gene or genes or epigenet* or correlat* or discriminat* or favo?rabl* or benefi* or positive or negative or identif* or baseline or heterogen*)).tw,kf. | 653939 |
| 25 | ((immun* or tumo?r) adj (factor* or feature or features or parameter* or variables or signature)).tw,kf. | 23874 |
| 26 | ((baseline adj2 characteristic*) or ((continuous or binary) adj2 variable*)).tw,kf. | 26403 |
| 27 | (responder* or (respon* and nonrespon*)).tw,kf. | 56365 |
| 28 | ((identif* or select* or discriminat*) adj6 (patients or subgroup* or group) adj12 benefit*).tw. | 13176 |
| 29 | ((likely or unlikely or associat* or correlat* or relation* or correspond* or discriminat*) adj6 benefit*).tw. | 27177 |
| 30 | (independent* adj6 (prognos* or predict* or associat* or factor* or variable* or parameter* or role* or risk or cohort* or validat*)).tw,kf. | 268490 |
| 31 | ((relationship or (associat* not associated-antigen-4*) or "associated with" or association* or correlat* or predict*) adj9 (remission* or ((objective or clinical or treatment or partial or complete or overall) adj3 (respons* or benefit* or activity or efficac* or efficiency or succes* or outcome*)) or CR or response rate* or disease control or surviv* or mortalit* or (death* not programmed death*) or ((fatal or lethal or patient) adj3 outcome*) or PFS or OS or ORR or ORRs or relaps* or recur* or refractor* or resistan* or fail* or progress*)).tw. | 654543 |
| **32** | **or/19-31 [biomarkers/predictive factors]** | **3191341** |
| **33** | **32 and 16 [ I secondary studies on melanoma + immune checkpoint inhibitors + biomarkers/predictive facors]** | **31** |
| **34** | **32 and 18 [ I primary studies on melanoma + immune checkpoint inhibitors + biomarkers/predictive facors]** | **400** |
| 35 | L-lactate dehydrogenase/ or exp chemokines, CXC/ or exp chemokines, CC/ or "inducible T-cell co-stimulator protein"/ or exp S100 proteins/ or Vascular Endothelial Growth Factors/ or Nitric Oxide/ or HMGB1 protein/ or exp Major Histocompatibility Complex/ or exp Genes, Tumor Suppressor/ or exp Tumor Suppressor Proteins/ | 352457 |
| 36 | exp cell count/ or tumor microenvironment/ or neoplastic cells, circulating/ or lymphocytes, tumor-infiltrating/ or lymphocytes/ or T-lymphocytes, regulatory/ or myeloid cells/ or neutrophils/ or eosinophils/ or monocytes/ | 456118 |
| 37 | genes, T-cell receptor/ or exp transcription factors/ or RNA, messenger/ or exp gene expression profiling/ or exp genetic association studies/ or microarray analysis/ or gene expression/ or transcriptome/ or gene expression regulation/ or down-regulation/ or gene expression regulation, neoplastic/ or transcriptional activation/ or up-regulation/ or genomics/ or exome/ or genomic library/ or genotype/ or haplotypes/ or exp sequence analysis/ or exp base sequence/ or genetic variation/ or antigenic variation/ or genetic heterogeneity/ or polymorphism, genetic/ or polymorphism, single nucleotide/ | 1973777 |
| 38 | (PLA2G7 or Lp-PLA2 or platelet-activating factor acetylhydrolas* or LDL-PLA2 or CTSD or cathepsin-D or TXNR* or Trxr1 or GRIM-12 or IRAK3 or IRAKM or IRAK-M or interleukin-1 receptor-associated kinase* or CD66* or CD-66* or CEACAM* or C-CAM* or CEA-related cell adhesion molecule 1).tw,kf,rn. | 10821 |
| 39 | (lactate dehydrogenas* or LDH or CXC* or (CC* and (chemokine* or lymphokin*)) or TARC or MHC* or Major Histocompatibility or nitric oxide* or HMGB1* or S-100 or S100 or S-100B or S100B or vascular endothelial growth factor* or VEGF* or GADD* or CHOP or DDIT3 or C-EBP or ICOS* or transcription factor* or (inducible adj2 (costimulator or co-stimulator or transcript* or protein*)) or CD278 or T cell receptor* or TCR or NY-ESO-1 or cancer testis-antigen or CTAG1* or PTEN).tw,kf. | 586816 |
| 40 | (((blood or myeloid or neutrophil* or eosinophil* or lymphocyte* or CD8* or CD4* or monocyte* or macrophage* or peripheral) adj3 (ratio or ratios or frequenc* or count or counts or number*)) or ANC or dLNR or AMC or AEC or RLC).tw,kf. | 144525 |
| 41 | (MDSC* or moMDSC* or suppressor cell* or tumor-associated immunotype* or ((tumor* or tumour* or melanom* or lymphohistiocyt* or CD45R0) adj3 (infiltrat* or resident*)) or TIL* or ((intratumor* or intra-tumor*) adj (T-cell* or T effector*))).tw,kf. | 86786 |
| 42 | (immun?regulator* or immun*-regulator* or (regulatory adj3 (T-cell* or Tcell*)) or Treg* or T-reg*).tw,kf. | 40430 |
| 43 | ((PD-1* or PD1* or PDL1* or PD-L1* or PDL-1* or B7-H1 or B7H1 or leukotriene B4 receptor* or BL-T1 or BLT1 or gene or genes or genetic or RNA or mRNA or DNA or differentially) adj3 (express* or overexpres* or upregulat* or up-regulat* or negative)).tw,kf. | 620352 |
| 44 | (GWA or GWAs or genomewide or genome-wide or whole genom* or Affymetrix or microarray* or genechip* or ((micro or gene or mRNA or RNA or DNA or cDNA or CGH* or oligonucleotid*) adj2 (chip* or array*)) or transcriptom* or (transcript* adj2 profil*) or (subtract* adj2 (hybrid* or CDNA or RNA or mRNA)) or (mRNA* and differential display*) or exome* or DEGs or ((RNA or next generation) adj seq*) or high throughput or signature* or genomic* or phenotyp* or genotype* or polymorph*).tw,kf. | 1214128 |
| 45 | ((sequencing adj9 (PCR or polymerase chain)) or (gene* adj3 (panel* or profile*)) or CGP or CGPs or mutanom* or (mutat* adj6 (sequenc* or somatic or antigen* or load* or burden or accumulat* or profile*))).tw,kf. | 122285 |
| 46 | (((circulating or cell-free or serum or soluble or blood) adj6 (tumo?r or antitumo* or melanom* or DNA or cytokine* or lymphokine* or IL-17 or IL17 or CTLA4 or CTLA-4 or CD25 or CD-25)) or CTC or CTCs or ctDNA or sCTLA-4 or sCTLA4 or sMICA or sCD25 or sCD-25).tw,kf. | 98767 |
| 47 | morphom*.tw,kf. | 48547 |
| **48** | **or/35-47 [specific factors]** | **3717392** |
| 49 | "prognosis"/ or exp "regression analysis"/ or (correlat* or relationship* or predict* or prognos* or likelihood or (associat* not (associated-antigen-4* or (associated adj3 immune-related))) or "associated with" or association* or prognostic or logistic or regression or baseline).tw. | 6503097 |
| **50** | exp "survival"/ or exp "mortality"/ or mortality.fs. or exp "disease-free survival"/ or exp "survival analysis"/ or exp "remission induction"/ or exp "treatment outcome"/ or "treatment failure"/ or exp "disease progression"/ or exp "recurrence"/ or exp "Kaplan-Meier estimate"/ or (Kaplan-Meier* or treatment outcome* or clinical outcome* or mortalit* or fatal* or (death* not programmed death*) or surviv* or remiss* or response* or clinical activit* or clinical benefit* or treatment fail* or ((tumo?r* or cancer* or melanom* or disease) adj3 progress*) or relaps* or recurren* or refractory or CR or PFS or OS or ORR or ORRs).tw,kf. | **5182857** |
| **51** | **49 and 50 [prognostic/mortality-FILTER** to combine with known prognostic factors**]** | **2154460** |
| **52** | **48 and 51 [specific factors + prognosis/survival filter]** | **524578** |
| **53** | **16 and 52 [II secondary studies - specific factors]** | **6** |
| **54** | **33 or 53 [I II all secondary studies]** | **33** |
| **55** | **remove duplicates from 54 [I II all secondary studies - deduplicated]** | **32** |
| **56** | **18 and 52 [II primary studies - specific factors]** | **287** |
| **57** | **34 or 56 [I II all primary studies]** | **473** |
| **58** | **remove duplicates from 57 [I II all primary studies - deduplicated]** | **429** |

**Supplementary Table 2.** Included publications per biomarker category and type

| **Biomarker category** | **Biomarker type** | **CTLA-4 ICI** | **PD-1 ICI** |
| --- | --- | --- | --- |
| ***Blood biomarkers*** | | | |
| Blood cytology | Lymphocyte count | (7, 8, 20, 32-34, 36-38, 42, 50, 53, 56, 68, 71, 72) |  |
|  | Abs monocyte count | (22, 36, 42, 51) |  |
|  | Abs neutrophil count | (19, 20, 22, 33, 63, 72) |  |
|  | Abs eosinophil count | (22, 42) |  |
|  | Neutrophil-lymphocyte ratio | (16, 19, 20, 33, 72) |  |
|  | White blood cell count | (20, 22, 33, 56) |  |
|  | MDSC | (9, 22, 36, 42, 44) | (23, 66) |
|  | NK cells | (28, 51) |  |
| Blood soluble factors | LDH | (6-8, 15, 16, 20, 26, 32-38, 42, 46, 54, 56, 63, 64, 68, 72) | (17) |
|  | Albumin | (6, 16) |  |
|  | C reactive protein | (53, 54, 56, 68) |  |
|  | erythrocyte sedimentation rate | (34) |  |
|  | VEGF | (37, 59, 71) |  |
|  | circulating tumor DNA | (24) | (24) |
|  | S100 | (34) |  |
| Blood soluble immune factors | pro-inflammatory cytokines | (9, 37, 59, 63) |  |
|  | anti-inflammatory cytokines | (59) |  |
|  | soluble MICA/B | (37) |  |
|  | soluble CD25 | (26, 37) |  |
|  | LAG3 | (13, 26) |  |
|  | soluble CTLA4 | (40) |  |
| T cell responses/regulation | melanoma-specific CD8+ T cells | (70) | (67) |
|  | melanoma -specific CD4+ T cells | (70) |  |
|  | melanoma -specific antibodies | (70) |  |
|  | (Ki67+)EOMES+ T-cells | (65) |  |
|  | Bim+ T cells |  | (18) |
|  | TCR diversity | (45, 50) | (62) |
|  | gamma/delta T cells | (69) |  |
|  | regulatory T cells | (42, 47, 56) | (23) |
|  | CTLA4/PD1 gene expression | (48) |  |
|  | immune gene expression | (49) |  |
| ***Tumor tissue biomarkers*** | | | |
| Tumor genetics | BRAF mutation | (7, 31, 33, 35, 41, 50, 55, 56, 68) | (27, 31, 39) |
|  | NRAS mutation | (30, 31, 35, 41, 56) | (27, 30, 31) |
|  | CKIT mutation | (33) |  |
|  | BCRA2 mutation |  | (27) |
|  | mutational load | (12, 57, 64) | (27) |
|  | neoantigen load | (43, 57, 64) |  |
| Tumor tissue expression | PD-L1 | (13, 54) | (10, 13, 14, 23, 27, 30, 60-62, 67) |
|  | PD-L2 | (64) | (60) |
|  | MHC molecules | (64) | (27, 30) |
|  | miR-222 | (21) |  |
|  | IDO | (25) |  |
|  | CTLA4/PD1 gene expression | (64) | (27) |
|  | IPRES gene signature |  | (27) |
|  | immune gene expression | (29, 52, 54, 64) | (27) |
| Tumor-infiltrating cells | Lymphocytes | (21, 54) | (60) |
|  | CD8+ T cells | (13, 25, 51, 54, 63) | (13, 30, 60, 62) |
|  | CD4+ T cells | (13, 25, 28) | (13, 30, 62) |
|  | CD20+ B cells | (54, 58) | (60) |
|  | CD56+ NK cells | (51) |  |
|  | Macrophages | (13, 51) | (13) |
|  | Regulatory T cells (Treg) | (25, 51) |  |
|  | PD-1+ T cells |  | (13, 60, 62) |
|  | Activated T cells | (21, 25, 64) | (13, 62) |
| ***Genomic biomarkers*** | | | |
|  | CTLA-4 polymorphisms | (11, 25, 46) |  |
|  | immune activation genes | (25) |  |
|  | HLA class I, HLA-A alleles | (25) |  |
